# Correlation between the triglyceride-to-high-density lipoprotein cholesterol ratio and other unconventional lipid parameters with the risk of prediabetes and Type 2 diabetes in patients with coronary heart disease: a *RCSCD-TCM* study in China

**DOI:** 10.1186/s12933-022-01531-7

**Published:** 2022-06-03

**Authors:** Tong Yang, Yijia Liu, Lin Li, Yanchao Zheng, Yang Wang, Jinyu Su, Rongrong Yang, Mingchi Luo, Chunquan Yu

**Affiliations:** 1 Tianjin University of Traditional Chinese Medicine, No. 10 Poyang Lake Road, Wet Zone, Tuanbo New City, Jinghai District, Tianjin, 301617 China; 2 Second Teaching Hospital of Tianjin University of Traditional Chinese Medicine, No. 816, Truth Road, Hebei District, Tianjin, 300150 China

**Keywords:** Coronary heart disease, Lipid, TG/HDL-C, Prediabetes, Type 2 diabetes

## Abstract

**Objective:**

Type 2 diabetes mellitus (T2DM) is often accompanied by undiagnosed dyslipidemia. Research on the association of unconventional lipid markers with prediabetes (pre-DM) and T2DM simultaneously is limited in coronary heart disease (CHD) patients.

**Methods:**

This study included 28,476 patients diagnosed with CHD. Their lipid levels, including triglycerides (TG), total cholesterol (TC), high-density lipoprotein cholesterol (HDL-C), and low-density lipoprotein cholesterol (LDL-C), were measured, and non-traditional lipid parameters were calculated. The patients were divided into three groups based on the diabetic status including normoglycemic (NG), pre-DM, and T2DM. Multiple logistic regression was used to compare the association of TG/HDL-C and other non-traditional lipid parameters with pre-DM and T2DM. The tertiles of TG/HDL-C included T1 (TG/HDL-C < 1.10), T2 (1.10 ≤ TG/HDL-C ≤ 1.89) and T3 (TG/HDL-C > 1.89). Low and high TG/HDL-C was defined with sex-specific cutoff points.

**Results:**

Multiple logistic regression results showed that the non-traditional lipid parameters, including non-HDL-C, LDL-C/HDL-C, TC/HDL-C, non-HDL-C/HDL-C and TG/HDL-C, were all correlated with the risk of pre-DM and T2DM. Meanwhile TG/HDL-C showed the strongest correlation (odds ratio [OR]: 1.19; 95% confidence interval [CI] 1.16–1.23), (OR: 1.36; 95% CI 1.33–1.39). When dividing TG/HDL-C into tertiles, using T1 as a reference, T3 was observed to have the highest association with both pre-DM and T2DM (OR: 1.60; 95% CI 1.48–1.74), (OR: 2.79; 95% CI 2.60–3.00). High TG/HDL-C was significantly associated with pre-DM and T2DM (OR: 1.69; 95% CI 1.52–1.88), (OR: 2.85; 95% CI 2.60–3.12). The association of TG/HDL-C with T2DM and pre-DM existed across different sex, age, smoking, and drinking statuses.

**Conclusion:**

Elevated non-traditional lipid parameters were significantly associated with pre-DM and T2DM in CHD patients, especially TG/HDL-C. High TG/HDL-C was the risk factor with a strong correlation with the risk of pre-DM and T2DM.

## Background

Diabetes mellitus (DM) and coronary heart disease (CHD) are two chronic diseases that pose a huge public health burden [1]. CHDs are often accompanied by DM, possibly because both conditions occur with the same risk factors, such as abnormal inflammatory responses or abnormal lipid metabolism [2]. Moreover, as an essential risk factor for CHD, DM can exacerbate the progression of atherosclerosis, resulting in poor clinical outcomes [3, 4]. A 2022 study in China by Junning Fan et al. on 500,000 Chinese has shown that diseases, including CHD and DM, greatly portend the risk of mortality among Chinese adults [5]. The management of glucose metabolism in patients with CHD is therefore particularly important. However, abnormal blood glucose metabolism, including prediabetes (pre-DM) and DM, has become increasingly common; by 2045, more than 600 million people are estimated to develop pre-DM, and the same number will develop DM, according to the 2017 global estimates of DM prevalence and 2045 projections [6]. Besides, Asians are more prone to DM especially type 2 DM (T2DM) and other CHD complications, than Westerners due to various factors [7].

Dyslipidemia often accompanies abnormal glucose metabolism [8]. In addition to traditional lipid parameters, including triglycerides (TG), total cholesterol (TC), high-density lipoprotein cholesterol (HDL-C), and low-density lipoprotein cholesterol (LDL-C), non-traditional lipid parameters like TG/HDL-C, LDL-C/HDL-C, non-HDL-C, TC/HDL-C and non-HDL-C/HDL-C are all closely related to the occurrence and development of pre-DM and T2DM. The reason may be that excess cholesterol accumulation leads to β-cell dysfunction, thereby impairing glucose tolerance and affecting insulin secretion. In addition, islet cholesterol deposition may lead to increased islet amyloid polypeptide aggregation and increased islet amyloid formation, further deteriorating β-cell function and affecting glucose homeostasis [9–12]. More importantly, they are also considered more predictive of CHD than conventional lipid parameters. These non-traditional lipid parameters can provide more information about conventional parameters, are difficult to quantify risk information and can better reflect interactions between lipid components [13]. Of these, the TG/HDL-C has been recognized as a potential predictive marker of insulin resistance (IR), which is a key trigger for the development of T2DM. Increased TG/HDL-C ratios have been shown to indicate a greater risk of new-onset T2DM in some studies [14]. Additionally, past studies have extensively explored sex-specific cutoff points for TG/HDL-C, which classify participants as high or low IR and cardiovascular disease (CVD) risk [15, 16]. However, few studies have compared the strength of the association of TG/HDL-C and other non-traditional lipid parameters with the occurrence of pre-DM and T2DM in the Chinese CHD population.

Therefore, this study aimed to compare the association of TG/HDL-C and other non-traditional lipid parameters with pre-DM and T2DM in the Chinese CHD population, test the association of high TG/HDL-C based on sex-specific cutoff points with pre-DM and T2DM.

## Methods

### Subjects

**Fig. 1:**
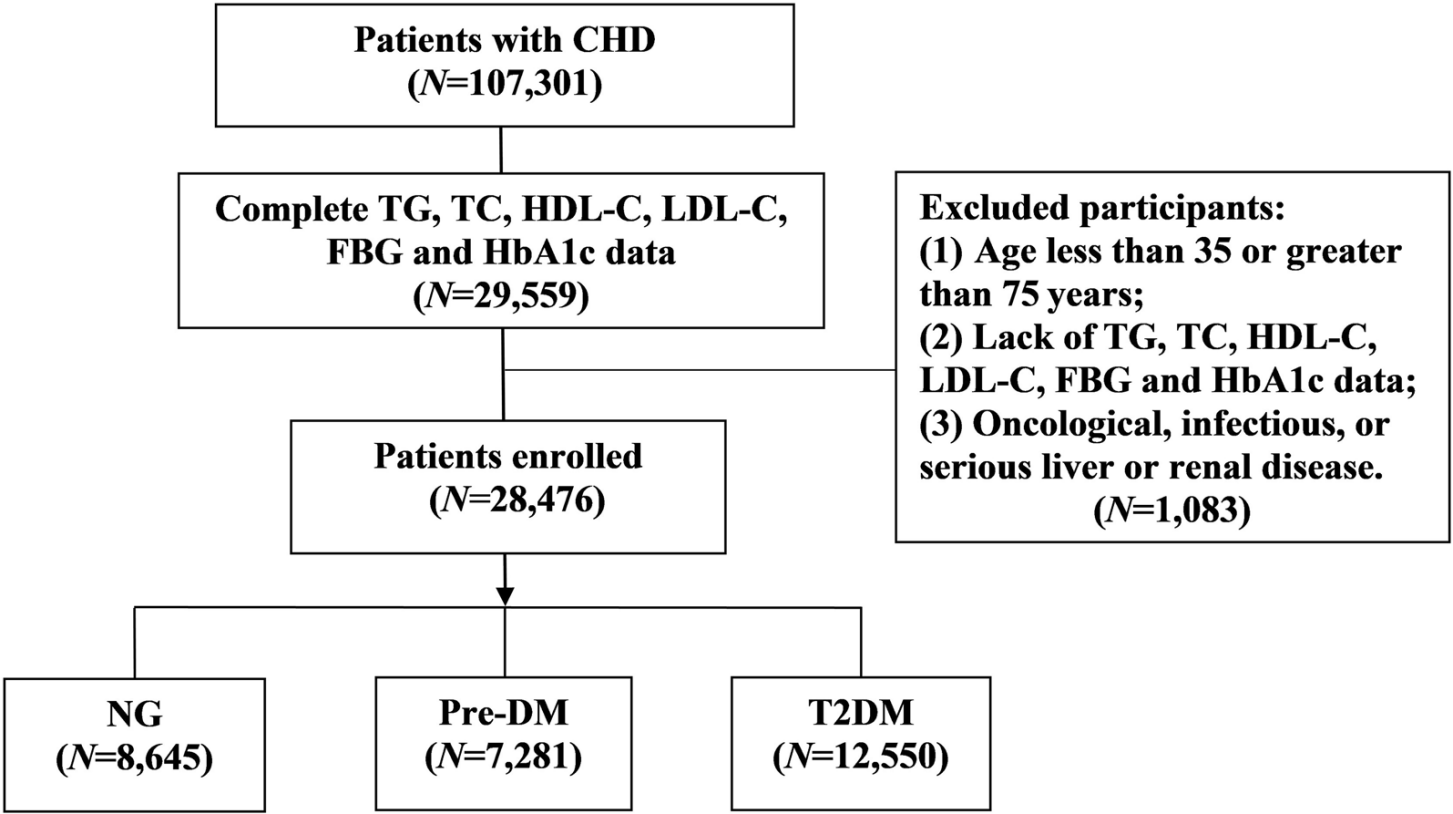
Flow chart of patient recruitment

We conducted a large, multicenter retrospective cohort study called *Retrospective Cohort Study on Adjuvant Treatment of Coronary Heart Disease Angina Pectoris with Chinese Patent Medicine (RCSCD-TCM)*. During the study, we established a CHD retrospective database, which included 107,301 inpatients with CHD from 6 hospitals in Tianjin, including Tianjin Chest Hospital, Tianjin Hospital of ITCWM Nankai Hospital, Tianjin Academy of Traditional Chinese Medicine Affiliated Hospital, First Teaching Hospital of Tianjin University of Traditional Chinese Medicine, Second Teaching Hospital of Tianjin University of Traditional Chinese Medicine, Tianjin Medical University General Hospital from January 1, 2014 to September 30, 2020. The following patients were excluded: (1) those younger than 35 years or older than 75 years; (2) those with oncological, infectious, or serious liver or renal diseases; (3) those who lacked TG, TC, HDL-C, LDL-C, fasting blood glucose (FBG) and hemoglobin A1c (HbA1c) data. Ultimately, 28,476 eligible subjects were enrolled in the final analysis. The flow chart of patient recruitment is shown in Fig. 1. The study was approved by the Ethics Committee of Tianjin University of Traditional Chinese Medicine (approval number TJUTCM-EC20190008) and certified by the Chinese Clinical Trials Registry on July 14, 2019 (registration number ChiCTR-1900024535) and on July 18, 2019, by ClinicalTrials.gov (registration number NCT04026724) [17].

### Data collection

The following data for this study analysis were acquired from the CHD retrospective database, where data came from medical records: clinical history, anthropometric data, blood analysis, and medical imaging data. Anthropometric data, including blood pressure, and personal information such as age, sex, smoking status, drinking status, family history of DM, current antihypertensive medication, and current anti-lipid medication were recorded. Fasting venous blood samples were obtained from all subjects on the second day of hospitalization. TG, TC, LDL-C, HDL-C, FBG and HbA1c were measured directly by an automatic hematology analyzer. The laboratory carries out quality control according to standard procedures. Non-HDL-C = TC – HDL-C; Non-HDL-C/HDL-C =  (TC – HDL-C)/HDL-C.

### Definitions

Smokers smoke at least 100 cigarettes in their lifetime [18]. Drinkers are defined as consuming alcohol at least 1 time per week [19]. Hypertension was defined as systolic blood pressure (SBP) ≥ 140 mmHg and/or diastolic blood pressure (DBP) ≥ 90 mmHg, or current use of antihypertensive medication [20]. Non-HDL-C = TC-HDL-C; hyperlipidemia was defined as TC ≥ 6.2 mmol/L, TG ≥ 2.3 mmol/L, LDL-C ≥ 4.1 mmol/L, or HDL-C ≤ 1.0 mmol/L [21]. Diabetic status includes normoglycemic (NG) (FBG5.6 < mmol/L or HbA1c < 5.7%), Pre-DM (5.6 ≤ FBG ≤ 6.9 mmol/L or 5.7 ≤ HbA1c ≤ 6.4%), T2DM (FBG ≥ 7.0 mmol/L or HbA1c ≥ 6.5%) [22]. According to the sex-specific cutoff of TG/HDL-C for identifying the risks of IR and CVDs, high TG/HDL-C was defined as TG/HDL-C > 2.5 in females and TG/HDL-C > 3.5 in males [15].

### Statistical analysis

The tertiles of TG/HDL-C were T1 (TG/HDL-C < 1.10), T2 (1.10 ≤ TG/HDL-C ≤ 1.89) and T3 (TG/HDL-C > 1.89). The Kolmogorov–Smirnov test was used to test the data for normality. Normally distributed continuous variables were presented as mean ± standard deviation, and non-normally distributed data were presented as median (interquartile). Demographic differences among groups were assessed using the Kruskal–Wallis H test. Categorical variables were expressed as counts and percentages (%), and differences between groups were examined with the chi-square test. Logistic regression models, calculated using odds ratio (OR) and 95% confidence interval (CI), were used to investigate the association of pre-DM and T2DM with various lipid parameters. Two-sided *P* < 0.05 was considered statistically significant. The collinearity of different models was tested before logistic regression. Missing values were imputed using the multiple imputation method. All statistical analyses were performed using the statistical package for the social sciences version 24.0 (IBM Corp, New York, NY, USA).

## Result

### Subject characteristics

28,476 participants were included in this study, including 13,321 (46.8%) females, 15,155 (53.2%) males, with a median age of 64 years, and 18,660 (65.5%) were over 60 years old. 30.4% (8465), 25.6% (7281), and 44.1% (12,550) were NG, pre-DM, and T2DM, respectively. TG/HDL-C levels and high TG/HDL-C distribution were higher in pre-DM and T2DM than in NG. Baseline characteristics of participants according to diabetic status are shown in Table 1.

**Table 1:** Baseline clinical characteristics according to diabetic status

Characteristics	NG *N* = 8,645	Pre-DM *N* = 7,281	T2DM *N* = 12,550	*P-*value
Age (y)				0.004
≤ 60	3065 (35.5)	2394 (32.9)	4357 (34.7)	
> 60	5580 (64.5)	4887 (67.1)	8193 (65.3)	
Sex				< 0.001
Male	4560 (52.7)	3717 (51.1)	6878 (54.8)	
Female	4085 (47.3)	3564 (48.9)	5672 (45.2)	
SBP (mmHg)	140.0 (125.0,153.3)	140.0 (125.9,154.8)	140.0 (126.0,155.0)	0.001
DBP (mmHg)	82.3 (75.6,90.0)	82.0 (75.8,90.0)	81.6 (75.9,90.0)	0.210
Drinking (%)	4572 (52.9)	3748 (51.5)	6698 (53.4)	0.034
Smoking (%)	3739 (43.3)	3987 (41.0)	5331 (42.5)	0.017
Hypertension (%)	4173 (48.3)	3654 (50.2)	6305 (50.2)	0.010
Hyperlipidemia (%)	1640 (19.0)	1397 (19.2)	2374 (18.9)	0.893
HbA1c (%)	5.8 (5.5,6.2)	6.2 (5.7,6.8)	7.5 (6.5,8.8)	< 0.001
FBG (mmol/L)	5.0 (4.7,5.3)	6.2 (5.9,6.5)	9.0 (7.7,11.6)	< 0.001
TG (mmol/L)	1.3 (1.0,1.8)	1.5 (1.1,2.0)	1.6 (1.2,2.3)	< 0.001
TC (mmol/L)	4.4 (3.7,5.2)	4.5 (3.7,5.2)	4.5 (3.7,5.3)	< 0.001
HDL-C (mmol/L)	1.1 (0.9,1.3)	1.1 (0.9,1.3)	1 (0.8,1.2)	< 0.001
LDL-C (mmol/L)	2.7 (2.1,3.4)	2.8 (2.1,3.4)	2.8 (2.1,3.4)	0.004
Non-HDL-C (mmol/L)	3.3 (2.6,4)	3.3 (2.7,4.1)	3.4 (2.7,4.2)	< 0.001
LDL-C/HDL-C	2.5 (1.9,3.2)	2.6 (2.0,3.3)	2.7 (2.1,3.5)	< 0.001
TC/HDL-C	4.0 (3.3,4.9)	4.1 (3.4,5.1)	4.4 (3.6,5.4)	< 0.001
Non-HDL-C/HDL-C	3.0 (2.3,3.9)	3.1 (2.4,4.1)	3.4 (2.6,4.4)	< 0.001
TG/HDL-C	1.2 (0.8,1.9)	1.4 (0.9,2.1)	1.6 (1.1,2.6)	< 0.001
High TG/HDL-C	675 (7.8)	902 (12.4)	2364 (18.8)	< 0.001
TG/HDL-C tertiles				< 0.001
T1	3624 (41.9)	2544 (34.9)	3371 (26.9)	
T2	2952 (34.1)	2498 (34.3)	4040 (32.2)	
T3	2069 (23.9)	2239 (30.8)	5139 (40.9)	
Family history of DM (%)	539 (6.2)	545 (7.5)	1682 (13.4)	< 0.001
Current antihypertensive medication (%)	5434 (62.9)	4732 (65.0)	8056 (64.2)	0.017
Current antilipidemic medication (%)	5229 (60.5)	4455 (61.2)	7302 (58.2)	< 0.001

Data are presented as median (interquartile) or number (proportion, %)

NG: normoglycemic; Pre-DM: pre-diabetes; T2DM: type 2 diabetes; SBP: systolic blood pressure; DBP: diastolic blood pressure; HbA1c: hemoglobin A1c; FBG: fasting blood glucose; TG: triglycerides; TC: total cholesterol; HDL-C: high-density lipoprotein cholesterol; LDL-C: low-density lipoprotein cholesterol; DM: diabetes

### Associations between pre-DM and T2DM with univariate

Univariate analysis results showed that age, sex, SBP, hypertension, family history of DM, current antihypertensive medication, TG, TC, HDL-C, LDL-C, non-HDL-C, LDL-C/HDL-C, TC/HDL-C, non-HDL-C/HDL-C, TG/HDL-C, high TG/HDL-C were associated with pre-DM and T2DM. Among non-traditional lipid parameters, TG/HDL-C was the highest risk factor associated with pre-DM and T2DM in CHD patients (OR: 1.19; 95% CI 1.16–1.23), (OR: 1.36; 95% CI 1.33–1.39) (Table 2).

**Table 2:** Associations between pre-DM and T2DM with univariate

Variables	Pre-DM			T2DM		
	OR	95% CI	*P-*value	OR	95% CI	*P-*value
Age	0.99	0.98–0.99	< 0.001	0.96	0.95–0.96	< 0.001
Sex						
Female	Reference			Reference		
Male	0.93	0.88–0.99	0.033	1.81	1.03–1.15	0.003
SBP	1.00	1.00–1.00	0.032	1.00	1.00–1.00	< 0.001
DBP	1.00	1.00–1.00	0.757	1.00	1.00–1.00	0.232
Drinking						
No	Reference			Reference		
Yes	0.95	0.89–1.00	0.076	1.02	0.97–1.07	0.487
Smoking						
No	Reference			Reference		
Yes	0.91	0.86–0.97	0.005	0.97	0.92–1.02	0.264
Hypertension						
No	Reference			Reference		
Yes	1.08	1.01–1.15	0.016	1.08	1.02–1.14	0.005
Hyperlipidemia						
No	Reference			Reference		
Yes	1.01	0.94–1.10	0.729	1.00	0.93–1.07	0.921
Family history of DM						
No	Reference			Reference		
Yes	1.22	1.08–1.38	0.002	2.33	2.10–2.58	< 0.001
Current antilipidemic medication						
No	Reference			Reference		
Yes	1.03	0.97–1.10	0.367	0.91	0.86–0.96	0.001
Current antihypertensive medication						
No	Reference			Reference		
Yes	1.10	1.03–1.17	0.005	1.06	1.00–1.12	0.047
TG	1.27	1.22–1.31	< 0.001	1.47	1.43–1.52	< 0.001
TC	1.05	1.03–1.07	0.001	1.07	1.05–1.09	< 0.001
HDL-C	0.92	0.84–0.97	0.007	0.41	0.38–0.44	< 0.001
LDL-C	1.05	1.01–1.08	0.007	1.05	1.02–1.08	< 0.001
Non-HDL-C	1.06	1.03–1.09	< 0.001	1.15	1.12–1.17	< 0.001
LDL-C/HDL-C	1.10	1.06–1.13	< 0.001	1.26	1.22–1.29	< 0.001
TC/HDL-C	1.10	1.08–1.13	< 0.001	1.27	1.25–1.30	< 0.001
Non-HDL-C/HDL-C/HDL-C	1.10	1.08–1.13	< 0.001	1.27	1.25–1.30	< 0.001
TG/HDL-C	1.19	1.16–1.23	< 0.001	1.36	1.33–1.39	< 0.001
TG/HDL-C						
Low	Reference			Reference		
High	1.67	1.50–1.86	< 0.001	2.74	2.50–3.00	< 0.001

OR: odds ratios; CI: confidence interval

### Associations between pre-DM and T2DM with traditional lipid parameters and non-traditional lipid parameters

As shown in Table 3, after adjusting for confounding factors, elevated TG (OR: 1.29; 95% CI 1.24–1.33) (OR: 1.50; 95% CI 1.45–1.55), TC (OR: 1.04; 95% CI 1.01–1.07) (OR: 1.08; 95% CI 1.06–1.11), and LDL-C (OR: 1.04; 95% CI 1.01–1.08) (OR: 1.06; 95% CI 1.03–1.10) in traditional lipid parameters were all associated with the risk of pre-DM and T2DM, with TG showing the highest association. Conversely, HDL-C may be a protective factor for pre-DM and T2DM (OR: 0.86; 95% CI 0.78–1.00) (OR: 0.39; 95% CI 0.35–0.43). The non-traditional lipid parameters non-HDL-C (OR: 1.06; 95% CI 1.03–1.09) (OR: 1.16; 95% CI 1.13–1.19), LDL-C/HDL-C (OR: 1.11; 95% CI 1.07–1.14) (OR: 1.27; 95% CI 1.23–1.30), TC/HDL-C (OR: 1.11; 95% CI 1.08–1.13) (OR: 1.28; 95% CI 1.25–1.30) and non-HDL-C/HDL-C (OR: 1.11; 95% CI 1.08–1.13) (OR: 1.28; 95% CI 1.25–1.30) were positively correlated with the risk of pre-DM and T2DM. TG/HDL-C remained the highest risk factor associated with pre-DM or T2DM in patients with CHD (OR: 1.21; 95% CI 1.16–1.25) (OR: 1.35; 95% CI 1.30–1.39).

**Table 3:** Associations between pre-DM and T2DM with traditional lipid parameters and non-traditional lipid parameters

Variables	Model 1^a^		Model 2^b^	
	Pre-DM	T2DM	Pre-DM	T2DM
	OR (95% CI)	OR (95% CI)	OR (95% CI)	OR (95% CI)
Traditional lipid parameter				
TG	1.27 (1.22–1.31) ^**^	1.47 (1.43–1.52) ^**^	1.29 (1.24–1.33) ^**^	1.50 (1.45–1.55) ^**^
TC	1.05 (1.03–1.07) ^*^	1.07 (1.05–1.09) ^**^	1.04 (1.01–1.07) ^*^	1.08 (1.06–1.11) ^**^
HDL-C	0.92 (0.84–0.97) ^*^	0.41 (0.38–0.44) ^**^	0.86 (0.78–1.00) ^*^	0.39 (0.35–0.43) ^**^
LDL-C	1.05 (1.01–1.08) ^*^	1.05 (1.02–1.08) ^**^	1.04 (1.01–1.08) ^*^	1.06 (1.03–1.10) ^**^
Untraditional lipid parameter				
Non-HDL-C	1.06 (1.03–1.09) ^**^	1.15 (1.12–1.17) ^**^	1.06 (1.03–1.09) ^**^	1.16 (1.13–1.19) ^**^
LDL-C/HDL-C	1.10 (1.06–1.13) ^**^	1.26 (1.22–1.29) ^**^	1.11 (1.07–1.14) ^**^	1.27 (1.23–1.30) ^**^
TC/HDL-C	1.10 (1.08–1.13) ^**^	1.27 (1.25–1.30) ^**^	1.11 (1.08–1.13) ^**^	1.28 (1.25–1.30) ^**^
Non-HDL-C/HDL-C	1.10 (1.08–1.13) ^**^	1.27 (1.25–1.30) ^**^	1.11 (1.08–1.13) ^**^	1.28 (1.25–1.30) ^**^
TG/HDL-C	1.19 (1.16–1.23) ^**^	1.36 (1.33–1.39) ^**^	1.21 (1.16–1.25) ^**^	1.35 (1.30–1.39) ^**^

^a^Model 1: unadjusted;

^b^Model 2: adjusted for age, sex, SBP, smoking, hypertension, family history of DM, current antilipidemic medication, current antihypertensive medication

Compared with NG, ^*^P < 0.05, ^**^P < 0.01

### Associations of pre-DM and T2DM with TG/HDL-C

As shown in Table 4, in Model 2, after adjusting for confounders, the results of multiple logistic regression analysis showed that when dividing TG/HDL-C into tertiles, using T1 as a reference, T3 was observed to have the highest association with both pre-DM and T2DM (OR: 1.60; 95% CI 1.48–1.74), (OR: 2.79; 95% CI 2.60–3.00). Compared with pre-DM, the association of TG/HDL-C with the risk of T2DM was stronger. When TG/HDL-C was used as a continuous variable in both unadjusted and adjusted models, the TG/HDL-C (tertiles) was consistent with the P for the trend of the pre-DM and T2DM (*P*_trend_ < 0.001). High TG/HDL-C was significantly associated with pre-DM and T2DM (OR: 1.69; 95% CI 1.52–1.88), (OR: 2.85; 95% CI 2.60–3.12).

**Table 4:** Associations between pre-DM and T2DM with TG/HDL-C

Variables	Model 1^a^		Model 2^b^	
	Pre-DM	T2DM	Pre-DM	T2DM
	OR (95% CI)	OR (95% CI)	OR (95% CI)	OR (95% CI)
TG/HDL-C				
T1	Reference	Reference	Reference	Reference
T2	1.21 (1.12–1.30) ^**^	1.47 (1.38–1.57) ^**^	1.22 (1.14–1.32) ^**^	1.50 (1.40–1.61) ^**^
T3	1.54 (1.43–1.67) ^**^	2.67 (2.49–2.86) ^**^	1.60 (1.48–1.74) ^**^	2.79 (2.60–3.00) ^**^
* P*_trend_	< 0.001	< 0.001	< 0.001	< 0.001
Low	Reference	Reference	Reference	Reference
High	1.67 (1.50–1.86) ^**^	2.74 (2.50–3.00) ^**^	1.69 (1.52–1.88) ^**^	2.85 (2.60–3.12) ^**^

T1: TG/HDL-C < 1.10; T2: 1.10 ≤ TG/HDL-C ≤ 1.89; T3:TG/HDL-C > 1.89

^a^Model 1: unadjusted;

^b^Model 2: adjusted for age, sex, SBP, smoking, hypertension, family history of DM, current antilipidemic medication, current antihypertensive medication

Compared with NG, ^**^P < 0.01

As shown in Table 5, after multivariate adjustment, TG/HDL-C was associated with pre-DM and T2DM in both sexes. But the association between it and pre-DM and T2DM in females (OR: 1.27; 95% CI 1.21–1.33) (OR: 1.49; 95% CI 1.44–1.56) was greater than in males (OR: 1.17; 95% CI 1.13–1.21) (OR: 1.30; 95% CI 1.26–1.34). As shown in Table 6, after adjusting for confounders, it was significantly associated with pre-DM and T2DM at different ages. When TG/HDL-C was used as a continuous variable, this association was greater in patients with CHD over age 60 (OR: 1.23; 95% CI 1.18–1.28) (OR: 1.44; 95% CI 1.39–1.49). Tables 7 and 8 showed that this association was significant across smoking and drinking after multivariate adjustmentc. When TG/HDL-C was used as a continuous variable, the association between it and pre-DM and T2DM in non-smokers (OR: 1.27; 95% CI 1.21–1.32) (OR: 1.46; 95% CI 1.40–1.51) and non-drinkers (OR: 1.24; 95% CI 1.19–1.29) (OR: 1.43; 95% CI 1.38–1.49) was greater. For different sexes, ages, smoking and drinking statuses, using T1 as a reference, T3 levels still presented the highest levels of pre-DM and T2DM risk, and high TG/HDL-C was significantly associated with pre-DM and DM.

**Table 5:** Associations between pre-DM and T2DM with TG/HDL-C according to sex

Variables	Model 1^a^		Model 2^b^	
	Pre-DM	T2DM	Pre-DM	T2DM
	OR (95% CI)	OR (95% CI)	OR (95% CI)	OR (95% CI)
TG/HDL-C				
Male				
Total	1.16 (1.12–1.19)^**^	1.28 (1.24–1.32)^**^	1.17 (1.13–1.21) ^**^	1.30 (1.26–1.34) ^**^
T1	Reference	Reference	Reference	Reference
T2	1.20 (1.08–1.34) ^**^	1.38 (1.26–1.51) ^**^	1.22 (1.09–1.35) ^**^	1.45 (1.32–1.60) ^**^
T3	1.51 (1.35–1.68) ^**^	2.37 (2.16–2.60) ^**^	1.54 (1.38–1.72) ^**^	2.56 (2.32–2.82) ^**^
*P*_trend_	< 0.001	< 0.001	< 0.001	< 0.001
Low	Reference	Reference	Reference	Reference
High	1.62 (1.37–1.90) ^**^	2.65 (2.31–3.05) ^**^	1.63 (1.38–1.93) ^**^	2.70 (2.35–3.11) ^**^
Female				
Total	1.26 (1.21–1.32) ^**^	1.50 (1.44–1.56) ^**^	1.27 (1.21–1.33) ^**^	1.49 (1.44–1.56) ^**^
T1	Reference	Reference	Reference	Reference
T2	1.22 (1.10–1.35) ^**^	1.57 (1.43–1.73) ^**^	1.22 (1.10–1.35) ^**^	1.54 (1.40–1.69) ^**^
T3	1.63 (1.45–1.83) ^**^	3.07 (2.77–3.41) ^**^	1.66 (1.47–1.86) ^**^	3.04 (2.74–3.38) ^**^
*P*_trend_	< 0.001	< 0.001	< 0.001	< 0.001
Low	Reference	Reference	Reference	Reference
High	1.69 (1.47–1.94) ^**^	2.91 (2.58–3.29) ^**^	1.72 (1.50–1.98) ^**^	2.91 (2.58–3.29) ^**^

^a^Model 1: unadjusted;

^b^Model 2: adjusted for age, SBP, smoking, hypertension, family history of DM, current antilipidemic medication, current antihypertensive medication

Compared with NG, ^**^P < 0.01

**Table 6:** Associations between pre-DM and T2DM with TG/HDL-C according to age

Variables	Model 1^a^		Model 2^b^	
	Pre-DM	T2DM	Pre-DM	T2DM
	OR (95% CI)	OR (95% CI)	OR (95% CI)	OR (95% CI)
TG/HDL-C				
≤ 60				
Total	1.18 (1.14–1.22) ^**^	1.30 (1.26–1.35) ^**^	1.18 (1.14–1.23) ^**^	1.31 (1.27–1.36) ^**^
T1	Reference	Reference	Reference	Reference
T2	1.33 (1.17–1.52) ^**^	1.51 (1.34–1.70) ^**^	1.34 (1.17–1.53) ^**^	1.53 (1.35–1.73) ^**^
T3	1.62 (1.42–1.84) ^**^	2.78 (2.48–3.13) ^**^	1.64 (1.44–1.88) ^**^	2.88 (2.56–3.25) ^**^
*P*_trend_	< 0.001	< 0.001	< 0.001	< 0.001
Low	Reference	Reference	Reference	Reference
High	1.70 (1.44–2.00) ^**^	2.99 (2.60–3.44) ^**^	1.72 (1.46–2.03) ^**^	3.06 (2.65–3.52) ^**^
> 60				
Total	1.22 (1.18–1.27) ^**^	1.44 (1.39–1.48) ^**^	1.23 (1.18–1.28) ^**^	1.44 (1.39–1.49) ^**^
T1	Reference	Reference	Reference	Reference
T2	1.16 (1.06–1.27) ^**^	1.46 (1.35–1.58) ^**^	1.17 (1.07–1.28) ^**^	1.49 (1.37–1.62) ^**^
T3	1.56 (1.42–1.73) ^**^	2.68 (2.46–2.93) ^**^	1.58 (1.43–1.75) ^**^	2.73 (2.50–2.99) ^**^
*P*_trend_	< 0.001	< 0.001	< 0.001	< 0.001
Low	Reference	Reference	Reference	Reference
High	1.68 (1.46–1.92) ^**^	2.61 (2.31–2.93) ^**^	1.65 (1.43–1.89) ^**^	2.66 (2.35–3.00) ^**^

^a^Model 1: unadjusted;

^b^Model 2: adjusted for sex, SBP, smoking, hypertension, family history of DM, current antilipidemic medication, current antihypertensive medication

Compared with NG, ^**^P < 0.01

**Table 7:** Associations between pre-DM and T2DM with TG/HDL-C according to smoking status

Variables	Model 1 ^a^		Model 2 ^b^	
	Pre-DM	T2DM	Pre-DM	T2DM
	OR (95% CI)	OR (95% CI)	OR (95% CI)	OR (95% CI)
TG/HDL-C				
Yes				
Total	1.15 (1.11–1.18) ^**^	1.29 (1.25–1.33) ^**^	1.16 (1.11–1.20) ^**^	1.30 (1.26–1.34) ^**^
T1	Reference	Reference	Reference	Reference
T2	1.18 (1.05–1.33) ^*^	1.37 (1.24–1.52) ^**^	1.20 (1.06–1.34) ^*^	1.43 (1.28–1.59) ^**^
T3	1.43 (1.27–1.62) ^**^	2.50 (2.26–2.78) ^**^	1.47 (1.30–1.66) ^**^	2.63 (2.35–2.92) ^**^
*P*_trend_	< 0.001	< 0.001	< 0.001	< 0.001
Low	Reference	Reference	Reference	Reference
High	1.59 (1.34–1.88) ^**^	2.82 (2.44–3.26) ^**^	1.62 (1.36–1.92) ^**^	2.84 (2.45–3.29) ^**^
No				
Total	1.25 (1.21–1.30) ^**^	1.44 (1.39–1.50) ^**^	1.27 (1.21–1.32) ^**^	1.46 (1.40–1.51) ^**^
T1	Reference	Reference	Reference	Reference
T2	1.23 (1.12–1.35) ^**^	1.55 (1.42–1.60) ^**^	1.24 (1.12–1.36) ^**^	1.55 (1.42–1.70) ^**^
T3	1.66 (1.50–1.85) ^**^	2.84 (2.59–3.12) ^**^	1.72 (1.54–1.91) ^**^	2.91 (2.65–3.20) ^**^
*P*_trend_	< 0.001	< 0.001	< 0.001	< 0.001
Low	Reference	Reference	Reference	Reference
High	1.71 (1.50–1.95) ^**^	2.69 (2.39–3.02) ^**^	1.74 (1.52–2.00) ^**^	2.83 (2.51–3.18) ^**^

^a^Model 1: unadjusted;

^b^Model 2: adjusted for age, sex, SBP, hypertension, family history of DM, current antilipidemic medication, current antihypertensive medication

Compared with NG, ^*^P < 0.05, ^**^P < 0.01

**Table 8:** Associations between pre-DM and T2DM with TG/HDL-C according to drinking status

Variables	Model 1^a^		Model 2^b^	
	Pre-DM	T2DM	Pre-DM	T2DM
	OR (95% CI)	OR (95% CI)	OR (95% CI)	OR (95% CI)
TG/HDL-C				
Yes				
Total	1.18 (1.14–1.22) ^**^	1.32 (1.28–1.36) ^**^	1.18 (1.14–1.23) ^**^	1.31 (1.27–1.36) ^**^
T1	Reference	Reference	Reference	Reference
T2	1.24 (1.12–1.37) ^**^	1.47 (1.34–1.61) ^**^	1.25 (1.12–1.38) ^**^	1.52 (1.38–1.66) ^**^
T3	1.57 (1.41–1.75) ^**^	2.52 (2.29–2.77) ^**^	1.60 (1.43–1.78) ^**^	2.66 (2.41–2.93) ^**^
*P*_trend_	< 0.001	< 0.001	< 0.001	< 0.001
Low	Reference	Reference	Reference	Reference
High	1.68 (1.45–1.95) ^**^	2.84 (2.50–3.22) ^**^	1.70 (1.46–1.97) ^**^	2.90 (2.55–3.29) ^**^
No				
Total	1.22 (1.17–1.27) ^**^	1.42 (1.37–1.47) ^**^	1.24 (1.19–1.29) ^**^	1.43 (1.38–1.49) ^**^
T1	Reference	Reference	Reference	Reference
T2	1.18 (1.06–1.31) ^**^	1.47 (1.34–1.62) ^**^	1.20 (1.08–1.33) ^*^	1.48 (1.34–1.63) ^**^
T3	1.54 (1.37–1.72) ^**^	2.87 (2.59–2.18) ^**^	1.62 (1.44–1.82) ^**^	2.94 (2.64–3.26) ^**^
*P*_trend_	< 0.001	< 0.001	< 0.001	< 0.001
Low	Reference	Reference	Reference	Reference
High	1.66 (1.43–1.92) ^**^	2.64 (2.32–3.00) ^**^	1.69 (1.46–1.97) ^**^	2.78 (2.44–3.17) ^**^

^a^Model 1: unadjusted;

^b^Model 2: adjusted for age, sex, SBP, smoking, hypertension, family history of DM, current antilipidemic medication, current antihypertensive medication

Compared with NG, ^*^P < 0.05, ^**^P < 0.01

## Discussion

This study investigated the correlation between TG.HDL-C and other unconventional lipid parameters with the risk of pre-DM and T2DM in Chinese patients with CHD. The results showed that non-traditional lipid parameters, especially TG/HDL-C, were related to the risk of pre-DM and T2DM. High TG/HDL-C, defined by the sex-specific TG/HDL-C cutoff point, is a risk factor for pre-DM and T2DM.

T2DM is a chronic metabolic disorder characterized by insufficient insulin production or IR caused by other factors [23]. Pre-DM is the intermediate stage between NG and DM, all people with T2DM pass the pre-DM stage, and about 5% to 10% of pre-DM will progress to T2DM each year [24]. Pre-DM and T2DM have been reported to be associated with an increased risk of CVD, including CHD [23, 25]. Therefore, managing pre-DM and T2DM risk factors is necessary. Glucose metabolism is closely related to lipid metabolism [26, 27]. Previous studies have demonstrated the correlation of lipid parameters including TG, TC, HDL-C, LDL-C, LDL-C/HDL-C, non-HDL-C, TC/HDL-C with pre-DM and DM [28–31]. Among them, TG and HDL-C have been considered important risk factors for developing CVD in Asians [32, 33]. The potential clinical significance of TG/HDL-C has been widely explored as a product of these two. Recent studies pointed out that TG/HDL-C was associated with IR and cardiometabolic disease risk; its cutoff for identifying risk differs between males and females [16, 34], suggesting that it may be a potential tool for identifying patients with DM. Consequently, we demonstrated its association with the risk of developing pre-DM and T2DM in the CHD population, and sex-specific high TG/HDL-C was a risk factor for pre-DM and T2DM.

The following reasons may explain the association of TG/HDL-C with pre-DM and T2DM: TG elevated results in increased free fatty acids (FFA), reduced insulin sensitivity [35], and continued exposure to FFA due to TG may reduce AMP-activated kinase protein activity and increase TG accumulation, leading to changes in pancreatic α-cell insulin signaling and hypersecretion of glucagon [36], thereby creating a vicious cycle between TG levels and IR. It leads to impaired glucose tolerance and the development of pre-DM and T2DM. At the same time, HDL protected β cells from cytokine- or glucose-induced apoptosis through two components, including ApoA1 (the major protein component of HDL) and S1P. Decreased HDL-C levels affect β-cell function or survival, which has a regulatory role in the pathogenesis of T2DM [37–39]. The combination of high TG and low HDL-C, known as atherogenic dyslipidemia, is also a strong risk factor for CHD. Therefore, the TG/HDL-C ratio was considered a potential predictive marker of IR and β-cell dysfunction. It is closely associated with pre-DM and T2DM as well as CVD development [40–42]. The study also verified that examined the association of TG/HDL-C with T2DM and pre-DM existed across different sex, age, smoking, and drinking statuses, as IR might changes with these factors [19, 43, 44]. Past studies have generally concluded that females exhibit more favorable metabolic risk profiles than males, including lower TG and higher HDL-C levels, and the association of dyslipidemia with DM appears to be stronger among males [45], middle-aged patients [46], and smokers and drinkers [47]. Conversely, when TG/HDL-C was used as a continuous variable in our study, it was associated with pre-DM and T2DM at different ages, sexes, smoking and drinking status, but stronger in females, people over 60 and those who do not smoke and drink alcohol. These differences might be due to the study population and sample size differences. Our study is aimed at CHD patients in China. Different races, different health conditions, and different sample sizes may affect the results of the study. A previous study in a Chinese population also showed that TG/HDL-C is not a marker of male IR but may be a marker of IR in Chinese non-obese females [48]. John Billimek et al. pointed out that although patients were prescribed similar lipid-lowering drug regimens, females with diabetes had worse lipid control than males [49]. Also, the anabolism is significantly lower in the elderly compared to middle-aged-onset patients. Elderly-onset T2DM patients have relatively preserved β-cell function and higher IR [14]. In addition, the relationship between alcohol consumption and IR T2DM remains controversial [47]. A meta-analysis evaluating the association between alcohol consumption and the risk of metabolic syndrome reported that compared with non-drinkers, very light drinkers were significantly associated with a reduction in the risk of metabolic syndrome. In contrast, heavy drinkers are associated with an increased risk of metabolic syndrome [50]. Further longitudinal studies may be needed for validation.

## Strengths and limitations

This present study has some strengths. First, based on our current knowledge, this was the largest population-based study of the association of non-traditional lipid parameters, especially TG/HDL-C, with pre-DM and T2DM in patients with CHD. The association of TG/HDL-C with pre-DM and T2DM was also verified at different ages, sex smoking and drinking statuses to exclude the influence of potential factors on this association. Secondly, possible confounders were also included in the analysis to rule out their interference with the results. Moreover, since a sex-specific cutoff point for TG/HDL-C was included in the core analysis, it may be beneficial to extend the clinical validation of this cutoff point in association with pre-DM and T2DM. Nonetheless, this study still had some limitations. Above all, as an observational study, this study was not suitable for examining the causal relationship between non-traditional lipid parameters and pre-DM and T2DM. Next, the current use of hypoglycemic agents and body mass index (BMI) as important confounders was not included in the regression model due to the missing data. We will conduct prospective cohort studies in the future to investigate causality and collect as comprehensive data as possible. Finally, there may be some unavoidable bias between centers being a multicenter study.

## Conclusions

In addition to traditionally determined lipid parameters, non-traditional lipid parameters were significantly correlated with pre-DM and T2DM in CHD patients, among which TG/HDL-C showed a stronger correlation. Early clinical lipid intervention is necessary, especially in CHD patients. Clinicians can take advantage of the potential value of the TG/HDL-C and its sex-specific cutoff points, which may serve as a simple and efficient dyslipidemia management tool for detecting and preventing the risk of DM in patients with CHD.

## Data Availability

The datasets used and/or analyzed in the current study are available from the corresponding author upon reasonable request.

## Abbreviations

DM: Diabetes mellitus
CHD: Coronary heart disease
Pre-DM: Prediabetes
T2DM: Type 2 DM
TG: Triglycerides
TC: Total cholesterol
HDL-C: High-density lipoprotein cholesterol
LDL-C: Low-density lipoprotein cholesterol
IR: Insulin resistance
FBG: Fasting blood glucose
HbA1c: Hemoglobin A1c
SBP: Systolic blood pressure
DBP: Diastolic blood pressure
NG: Normoglycemic
OR: Odds ratio
Cl: Confidence intervals
CVD: Cardiovascular disease
FFA: Free fatty acids; BMI: Body mass index

## References

[1] Konerding U, Redaèlli M, Ackermann K, Altin S, Appelbaum S, Biallas B, Bödecker AW, Botzenhardt S, Chermette C, Cichocki M, Dapper I, Dehnen K, Funke C, Gawlik A, Giesen L, Goetz J, Graf C, Hagen B, Heßbrügge M, Höhne PH, Kleinert J, Könnecke H, Küppers L, Kuth N, Lehmann L, Lendt C, Majjouti K, Nacak Y, Neuhausen A, Pilic L, Schneider L, Scholl M, Simic D, Sönnichsen A, Thielmann A, Van der Arend I, Vitinius F, Weltermann B, Wild D, Wilm S, Stock S (2021). A pragmatic randomised controlled trial referring to a Personalised Self-management SUPport Programme (P-SUP) for persons enrolled in a disease management programme for type 2 diabetes mellitus and/or for coronary heart disease. Trials.

[2] Xu W, Tian M, Zhou Y (2018). The relationship between insulin resistance, adiponectin and C-reactive protein and vascular endothelial injury in diabetic patients with coronary heart disease. Exp Ther Med.

[3] Babes EE, Bustea C, Behl T, Abdel-Daim MM, Nechifor AC, Stoicescu M, Brisc CM, Moisi M, Gitea D, Iovanovici DC, Bungau AF, Tit DM, Bungau SG (2022). Acute coronary syndromes in diabetic patients, outcome, revascularization, and antithrombotic therapy. Biomed Pharmacother.

[4] Dwivedi AK, Dubey P, Reddy SY, Clegg DJ (2022). Associations of glycemic index and glycemic load with cardiovascular disease: updated evidence from meta-analysis and cohort studies. Curr Cardiol Rep.

[5] Fan J, Sun Z, Yu C, Guo Y, Pei P, Yang L, Chen Y, Du H, Sun D, Pang Y, Zhang J, Gilbert S, Avery D, Chen J, Chen Z, Lyu J, Li L (2022). China Kadoorie Biobank Collaborative Group. Multimorbidity patterns and association with mortality in 0.5 million Chinese adults. Chin Med J.

[6] Yuan D, Jiang P, Zhu P, Jia S, Zhang C, Liu Y, Liu R, Xu J, Tang X, Zhao X, Gao R, Yang Y, Xu B, Gao Z, Yuan J (2021). Prognostic value of fibrinogen in patients with coronary artery disease and prediabetes or diabetes following percutaneous coronary intervention: 5-year findings from a large cohort study. Cardiovasc Diabetol.

[7] Zarkasi KA, Abdul Murad NA, Ahmad N, Jamal R, Abdullah N (2022). Coronary heart disease in type 2 diabetes mellitus: genetic factors and their mechanisms, gene-gene, and gene-environment interactions in the asian populations. Int J Environ Res Public Health.

[8] Zhao JV, Liu F, Schooling CM, Li J, Gu D, Lu X (2022). Using genetics to assess the association of commonly used antihypertensive drugs with diabetes, glycaemic traits and lipids: a trans-ancestry Mendelian randomisation study. Diabetologia.

[9] Ouchi G, Komiya I, Taira S, Wakugami T, Ohya Y (2022). Triglyceride/low-density-lipoprotein cholesterol ratio is the most valuable predictor for increased small, dense LDL in type 2 diabetes patients. Lipids Health Dis.

[10] Ye Y, Gao J, Liang J, Yang Y, Lv C, Chen M, Wang J, Zhu D, Rong R, Xu M, Zhu T, Yu M (2021). Association between preoperative lipid profiles and new-onset diabetes after transplantation in Chinese kidney transplant recipients: a retrospective cohort study. J Clin Lab Anal.

[11] Peng J, Zhao F, Yang X, Pan X, Xin J, Wu M, Peng YG (2021). Association between dyslipidemia and risk of type 2 diabetes mellitus in middle-aged and older Chinese adults: a secondary analysis of a nationwide cohort. BMJ Open.

[12] Bai Z, Zhang DS, Zhang R, Yin C, Wang RN, Huang WY, Ding J, Yang JL, Huang PY, Liu N, Wang YF, Cheng N, Bai YN (2021). A nested case-control study on relationship of traditional and combined lipid metabolism indexes with incidence of diabetes. Zhonghua Liu Xing Bing Xue Za Zhi.

[13] Zhu L, Lu Z, Zhu L, Ouyang X, Yang Y, He W, Feng Y, Yi F, Song Y (2015). Lipoprotein ratios are better than conventional lipid parameters in predicting coronary heart disease in Chinese Han people. Kardiol Pol.

[14] Kim J, Shin SJ, Kim YS, Kang HT (2021). Positive association between the ratio of triglycerides to high-density lipoprotein cholesterol and diabetes incidence in Korean adults. Cardiovasc Diabetol.

[15] Salazar MR, Carbajal HA, Espeche WG, Leiva Sisnieguez CE, Balbín E, Dulbecco CA, Aizpurúa M, Marillet AG, Reaven GM (2012). Relation among the plasma triglyceride/high-density lipoprotein cholesterol concentration ratio, insulin resistance, and associated cardio-metabolic risk factors in men and women. Am J Cardiol.

[16] Salazar MR, Carbajal HA, Espeche WG, Leiva Sisnieguez CE, March CE, Balbín E, Dulbecco CA, Aizpurúa M, Marillet AG, Reaven GM (2013). Comparison of the abilities of the plasma triglyceride/high-density lipoprotein cholesterol ratio and the metabolic syndrome to identify insulin resistance. Diab Vasc Dis Res.

[17] Li Z, He Y, Wang S, Li L, Yang R, Liu Y, Cheng Q, Yu L, Zheng Y, Zheng H, Gao S, Yu C (2022). Association between triglyceride glucose index and carotid artery plaque in different glucose metabolic states in patients with coronary heart disease: a RCSCD-TCM study in China. Cardiovasc Diabetol.

[18] Barua RS, Rigotti NA, Benowitz NL, Cummings KM, Jazayeri MA, Morris PB, Ratchford EV, Sarna L, Stecker EC, Wiggins BS (2018). 2018 ACC Expert Consensus Decision Pathway on Tobacco Cessation Treatment: A Report of the American College of Cardiology Task Force on Clinical Expert Consensus Documents. J Am Coll Cardiol.

[19] Ng R, Sutradhar R, Yao Z, Wodchis WP, Rosella LC (2020). Smoking, drinking, diet and physical activity-modifiable lifestyle risk factors and their associations with age to first chronic disease. Int J Epidemiol.

[20] Al-Makki A, DiPette D, Whelton PK, Murad MH, Mustafa RA, Acharya S, Beheiry HM, Champagne B, Connell K, Cooney MT, Ezeigwe N, Gaziano TA, Gidio A, Lopez-Jaramillo P, Khan UI, Kumarapeli V, Moran AE, Silwimba MM, Rayner B, Sukonthasan A, Yu J, Saraffzadegan N, Reddy KS, Khan T (2022). Hypertension pharmacological treatment in adults: a World Health Organization guideline executive summary. Hypertension.

[21] Grundy SM, Stone NJ, Bailey AL, Beam C, Birtcher KK, Blumenthal RS, Braun LT, de Ferranti S, Faiella-Tommasino J, Forman DE, Goldberg R, Heidenreich PA, Hlatky MA, Jones DW, Lloyd-Jones D, Lopez-Pajares N, Ndumele CE, Orringer CE, Peralta CA, Saseen JJ, Smith SC, Sperling L, Virani SS, Yeboah J (2019). 2018 AHA/ACC/AACVPR/AAPA/ABC/ACPM/ADA/AGS/APhA/ASPC/NLA/PCNA guideline on the management of blood cholesterol: a report of the American College of Cardiology/American Heart Association Task Force on Clinical Practice Guidelines. Circulation.

[22] Medina-Chávez JH, Vázquez-Parrodi M, Mendoza-Martínez P, Ríos-Mejía ED, de Anda-Garay JC, Balandrán-Duarte DA. Protocolo de Atención Integral: prevención, diagnóstico y tratamiento de diabetes mellitus 2 [Integrated Care Protocol: Prevention, diagnosis and treatment of diabetes mellitus 2]. Rev Med Inst Mex Seguro Soc. 2022; 60 (Supl 1):S4-S18. Spanish. **PMID: 35135039**. PMC10395976 35135039

[23] Yun JS, Ko SH (2021). Current trends in epidemiology of cardiovascular disease and cardiovascular risk management in type 2 diabetes. Metabolism.

[24] Tabák AG, Herder C, Rathmann W, Brunner EJ, Kivimäki M (2012). Prediabetes: a high-risk state for diabetes development. Lancet.

[25] Huang Y, Cai X, Mai W, Li M, Hu Y (2016). Association between prediabetes and risk of cardiovascular disease and all cause mortality: systematic review and meta-analysis. BMJ.

[26] Lauber C, Gerl MJ, Klose C, Ottosson F, Melander O, Simons K (2022). Lipidomic risk scores are independent of polygenic risk scores and can predict incidence of diabetes and cardiovascular disease in a large population cohort. PLoS Biol.

[27] Zhang M, Zhang J, Chua HZ, Feng R, Lu M, Tian Y (2021). Core outcome set for stable angina pectoris in traditional Chinese medicine (COS-SAP-TCM). Acupuncture Herbal Med..

[28] Alrasheed AA (2022). Dyslipidemia Among Patients With Type 1 Diabetes and Its Associated Factors in Saudi Arabia: An Analytical Cross-Sectional Study. Cureus.

[29] Wei L, Wei M, Chen L, Liang S, Gao F, Cheng X, Jiang H (2021). Low-density lipoprotein cholesterol : high-density lipoprotein cholesterol ratio is associated with incident diabetes in Chinese adults: a retrospective cohort study. J Diabetes Investig.

[30] Sunil B, Ashraf AP (2020). Dyslipidemia in pediatric type 2 diabetes mellitus. Curr Diab Rep.

[31] Ren X, Chen ZA, Zheng S, Han T, Li Y, Liu W, Hu Y (2016). Association between Triglyceride to HDL-C Ratio (TG/HDL-C) and Insulin Resistance in Chinese Patients with Newly Diagnosed Type 2 Diabetes Mellitus. PLoS ONE.

[32] American Diabetes Association (2017). Standards of medical care in diabetes-2017 abridged for primary care providers. Clin Diabetes.

[33] Gonzáles-Rubianes DZ, Figueroa-Osorio LK, Benites-Zapata VA, Pacheco-Mendoza J, Herrera-Añazco P (2022). Utility of TG/HDL-c ratio as a predictor of mortality and cardiovascular disease in patients with chronic kidney disease undergoing hemodialysis: a systematic review. Hemodial Int.

[34] Uruska A, Zozulinska-Ziolkiewicz D, Niedzwiecki P, Pietrzak M, Wierusz-Wysocka B (2018). TG/HDL-C ratio and visceral adiposity index may be useful in assessment of insulin resistance in adults with type 1 diabetes in clinical practice. J Clin Lipidol.

[35] Lai M, Fang F, Ma Y, Yang J, Huang J, Li N, Kang M, Xu X, Zhang J, Wang Y, Peng Y (2020). Elevated Midtrimester Triglycerides as a Biomarker for Postpartum Hyperglycemia in Gestational Diabetes. J Diabetes Res.

[36] Manell H, Kristinsson H, Kullberg J (2019). Hyperglucagonemia in youth is associated with high plasma free fatty acids, visceral adiposity, and impaired glucose tolerance. Pediatr Diabetes.

[37] Di Bartolo BA, Cartland SP, Genner S, Manuneedhi Cholan P, Vellozzi M, Rye KA, Kavurma MM (2021). HDL improves cholesterol and glucose homeostasis and reduces atherosclerosis in diabetes-associated atherosclerosis. J Diabetes Res.

[38] Sposito AC, de Lima-Junior JC, Moura FA, Barreto J, Bonilha I, Santana M, Virginio VW, Sun L, Carvalho LSF, Soares AAS, Nadruz W, Feinstein SB, Nofer JR, Zanotti I, Kontush A, Remaley AT (2019). Reciprocal multifaceted interaction between HDL (High-Density Lipoprotein) and Myocardial Infarction. Arterioscler Thromb Vasc Biol.

[39] Rütti S, Ehses JA, Sibler RA, Prazak R, Rohrer L, Georgopoulos S, Meier DT, Niclauss N, Berney T, Donath MY, von Eckardstein A (2009). Low- and high-density lipoproteins modulate function, apoptosis, and proliferation of primary human and murine pancreatic beta-cells. Endocrinology.

[40] Grundy SM (2006). Atherogenic dyslipidemia associated with metabolic syndrome and insulin resistance. Clin Cornerstone.

[41] Kannel WB, Vasan RS, Keyes MJ, Sullivan LM, Robins SJ (2008). Usefulness of the triglyceride-high-density lipoprotein versus the cholesterol-high-density lipoprotein ratio for predicting insulin resistance and cardiometabolic risk (from the Framingham Offspring Cohort). Am J Cardiol.

[42] Lim EL, Hollingsworth KG, Aribisala BS, Chen MJ, Mathers JC, Taylor R (2011). Reversal of type 2 diabetes: normalisation of beta cell function in association with decreased pancreas and liver triacylglycerol. Diabetologia.

[43] Ferrannini E, Natali A, Capaldo B, Lehtovirta M, Jacob S, Yki-Järvinen H (1997). Insulin resistance, hyperinsulinemia, and blood pressure: role of age and obesity European Group for the Study of Insulin Resistance (EGIR). Hypertension.

[44] Giha HA, AlDehaini DMB, Joatar FE, Ali ME, Al-Harbi EM, Al Qarni AA (2022). Hormonal and metabolic profiles of obese and nonobese type 2 diabetes patients: implications of plasma insulin, ghrelin, and vitamin D levels. Cardiovasc Endocrinol Metab.

[45] Ji XW, Feng GS, Li HL, Fang J, Wang J, Shen QM, Han LH, Liu DK, Xiang YB (2021). Gender differences of relationship between serum lipid indices and type 2 diabetes mellitus: a cross-sectional survey in Chinese elderly adults. Ann Transl Med.

[46] Zhang Y, Qin P, Lou Y, Zhao P, Li X, Qie R, Wu X, Han M, Huang S, Zhao Y, Liu D, Wu Y, Li Y, Yang X, Zhao Y, Feng Y, Wang C, Ma J, Peng X, Chen H, Zhao D, Xu S, Wang L, Luo X, Zhang M, Hu D, Hu F (2020). Association of TG/HDLC ratio trajectory and risk of type 2 diabetes: a retrospective cohort study in China. J Diabetes.

[47] Baek W, Lee JW, Lee HS, Han D, Choi SY, Chun EJ, Han HW, Park SH, Sung J, Jung HO, Lee H, Chang HJ (2021). Concurrent smoking and alcohol consumers had higher triglyceride glucose indices than either only smokers or alcohol consumers: a cross-sectional study in Korea. Lipids Health Dis.

[48] He J, He S, Liu K, Wang Y, Shi D, Chen X (2014). The TG/HDL-C Ratio Might Be a Surrogate for Insulin Resistance in Chinese Nonobese Women. Int J Endocrinol.

[49] Billimek J, Malik S, Sorkin DH, Schmalbach P, Ngo-Metzger Q, Greenfield S, Kaplan SH (2015). Understanding disparities in lipid management among patients with type 2 diabetes: gender differences in medication nonadherence after treatment intensification. Womens Health Issues.

[50] Sun K, Ren M, Liu D, Wang C, Yang C, Yan L (2014). Alcohol consumption and risk of metabolic syndrome: a meta-analysis of prospective studies. Clin Nutr.

